# Does An Education Seminar Intervention Improve the Parents’ Knowledge on Vaccination? Evidence from Yiwu, East China

**DOI:** 10.3390/ijerph120403469

**Published:** 2015-03-24

**Authors:** Yu Hu

**Affiliations:** 1 Institute of Immunization and Prevention, Zhejiang Center for Disease Control and Prevention, Hangzhou 310051, China; E-Mail: husix@163.com; 2 School of Public Health, Medical College, Zhejiang University, Hangzhou 310051, China

**Keywords:** vaccination, caregivers, education seminar, knowledge

## Abstract

*Background*: caregivers’ knowledge on vaccination is an important impact factor for their children’s vaccination status. The aims of this study were to evaluate the caregivers’ knowledge of vaccination, and to assess effectiveness of a health education seminar for improving caregivers’ knowledge on immunization. *Methods*: pre- and post-assessment design was adopted for a single group to evaluate the effectiveness of the health education seminar on vaccination. The seminar consisted of a lecture using simple understandable language. Improvements in total knowledge score before and after the seminar were assessed using a validated questionnaire that included ten questions. Description analysis and non-parametric tests were applied to evaluate and compare the vaccination knowledge level before and after the seminar. *Results*: 378 caregivers participated in this study. The majority were mothers. Of the ten questions, the correct response rates had significantly increased for nine questions after the education seminar. The mean total score of the assessment before the seminar was 5.2 ± 1.2 while that was 8.4 ± 0.9 for the assessment after the seminar, with a significant increase of 3.18 points. *Conclusion*: a short education seminar designed for caregivers had a remarkable effect on their vaccination knowledge. Health education on vaccination targeting migrant caregivers, caregivers with lower education level or household income, and employed caregivers are needed in future.

## 1. Introduction

Vaccination is regarded as one of the greatest public health achievements of the 20th century and one of the most cost-effective preventive services for children [1]. The substantial reduction in the incidence of vaccine preventable diseases (VPDs) makes caregivers have little or even no experience with these VPDs, hence, the benefits of immunization and the risk of not immunizing are not valued as much as they were at the beginning of the expanded program of immunization (EPI). For example, the success of immunization has made a measles case or a polio case very rare while there are constant reports on adverse events following immunization (AEFI) and concerns of vaccine safety. Caregivers’ decision on immunization would be negatively affected by paradoxical information or misinformation on vaccine safety from the media [2].

Vaccination hesitancy has become a subject of growing attention in the literature. Vaccination hesitant individuals have been defined as “a heterogeneous group in the middle of a continuum ranging from total acceptors to complete refusers” [3]. In a previous study [3], the three impact factors of vaccination hesitancy were found: contextual influences, individual or group influences (including knowledge and awareness), and vaccine or vaccination specific issues.

In China, EPI was introduced in 1978. The successes of Chinese EPI include eradication of smallpox and poliomyelitis, and substantial decrease in the incidence and mortality of vaccine-preventable diseases, such as hepatitis B, and measles. For example, in China, the hepatitis B virus surface antigen carrier rate has decreased from 8.75% (1979, before introduction of hepatitis B vaccine) to 7.18% (2006, after introduction of hepatitis B vaccine) [4]. The annual reported measles incidence rate has decreased from 1057.95 per 100,000 (1951–1965, before introduction of measles vaccine) to 10.62 per 100,000 (1986–2004, after introduction of measles vaccine) [5].

The success of EPI in China relies on a constantly high immunization coverage, which requires caregivers’ positive awareness of vaccination and willingness to immunize their children in turn. In our previous study, insufficient knowledge of immunization schedule was a significant risk factor of incomplete vaccination [6]. According to a review exploring the reasons for incomplete or no vaccinations [7], insufficient immunization knowledge of caregivers was the most common risk factor. Similarly, many studies [8,9,10,11] found caregivers’ lack of knowledge on vaccination was an obstacle that led to lower coverage.

Besides, failure to be vaccinated on time would increase the susceptible period of children, thus limiting herd immunity. This problem was well illustrated in a large measles epidemic in the USA [12], where delayed vaccination of measles containing vaccine (MCV) was identified as one of the main cause. The most common determinants on the timeliness of vaccination were the positive understanding and awareness of the importance of vaccination and following the recommended vaccination schedule [13]. It was also consistent with the study from China. For example, Han [14] found that parents who vaccinated their children on time had a higher level of immunization knowledge than those who delayed. The negative attitude towards vaccine safety was mainly due to a lack of knowledge of vaccination. Recently, there had been a shift from efforts to increase not only coverage but also the timeliness of vaccination. For example, the timeliness coverage of the first dose of measles containing vaccine was set at 95% since 2010 in Zhejiang province [15].

Educating caregivers whose children are likely not to be vaccinated was indicated as an effective method to improve immunization coverage through increasing their knowledge, attitude and practice towards immunization [16,17]. For example, Owais A [17] conducted a community-based randomized-controlled trial on health education intervention of immunization among 366 mother-infant pairs. After a three-month intervention period, they found the coverage for all three doses of hepatitis B vaccine was 72.1% and 51.7% in intervention group and control group, respectively. There were some reports of the evaluation of the impact on the health education intervention for increasing the parental knowledge of immunization worldwide [18,19,20,21,22]. These reports indicated that a health education program could improve the knowledge, attitude and practice towards vaccination among caregivers and suggested this strategy should be focused on the caregivers with lower education level or with misinformation/poor perception of immunization and should be integrated into the immunization program.

The aims of this study were: (1) to evaluate the knowledge of caregivers on immunization; (2) to evaluate effectiveness of a health education seminar for improving caregivers’ knowledge on immunization; and (3) to compare caregivers knowledge level across different demographic variables.

## 2. Methods

### 2.1. Study Design

Our study was implemented in Yiwu, which is a county in the center of Zhejiang province, east China, with an area of 1105.5 Km^2^ and a population of 1.9 million (2014 census). Immunization service is provided by 13 vaccination clinics for all eligible children in Yiwu. All 13 vaccination clinics participated in our study. Before the study began, the main researchers held a meeting with the vaccination personnel of all 13 vaccination clinics and described the purpose and details of the study. This study adopted a one group pre-assessment and post-assessment design to evaluate the influence of a health education seminar on vaccine and vaccination. This study was implemented for a two-month period from June to July, 2014. The health education seminar was held every week, usually on a Friday afternoon. In Totall, eight seminars were conducted during the study period.

### 2.2. Recruitment

Caregivers who visited any vaccination clinic and had a child younger than one year old and lived in Yiwu were invited to attend the health education seminar. The vaccination personnel in the vaccination clinics gave the purpose and content of the health education seminar to the eligible caregivers. Caregivers who were interested in attending the seminar were invited to register and to attend the seminar at the proposed time.

### 2.3. Content of the Health Education Seminar on Vaccination

The health education seminar on vaccination was developed by researchers from Zhejiang provincial center for disease control and prevention (CDC) and Yiwu CDC. It was delivered through a didactic lecture through a Powerpoint slide presentation. The content of the seminar was prepared to include issues on the importance of vaccination, the schedule of vaccination, immunization policy in China, immunization doses, AEFI, and contradictions. The content validity and understanding of the seminar were assessed by an expert panel, including three EPI management staff from Yiwu CDC and five vaccination personnel from vaccination clinics in Yiwu, and modifications were made to suit culture and context of the people of Yiwu. At the end of each lecture, the platform was open to caregivers to ask questions and to get their feedback. The seminar lasted approximately one hour. The outcomes of the seminar included knowing the EPI policy of China and the immunization schedules, knowing the basic knowledge about vaccines, underlying the diseases that could be prevented by vaccines and the importance of completeness of vaccination, the correct idea to weigh the disease as more serious than the AEFI, and the precautions of vaccination.

### 2.4. Survey Questionnaire

In order to achieve the objectives of this study, a questionnaire was designed to evaluate caregivers’ knowledge level on vaccination. We conducted a pilot test of the questionnaire in Yiwu. Ten eligible caregivers completed the questionnaires and commented on the questions, which were not included in the analysis. These ten questionnaires and the caregivers’ comments were discussed by the researchers and EPI management staff from Yiwu CDC and necessary amendments were made accordingly. A pre-assessment before the seminar was administered to caregivers who participated in our study while a post assessment after seminar was administered again to evaluate the influence of the intervention. The questionnaire included two parts: (1) socio-demographic variables of the surveyed caregivers, such as gender, age, immigration status, number of pre-school children, family size, employment status, education level, and household income; and (2) ten structured questions on knowledge on vaccination and all of the questions were closed ended (yes/no).

### 2.5. Statistical Analysis

We used description statistics to calculate the frequency or percentage of each socio-demographic variable and correct response rate of each knowledge question on vaccination. Scoring of the questions of vaccination knowledge was determined by giving one point for each correct answer while zero for each incorrect answer or no response. Thus, the maximum possible score was 10 if surveyed caregiver chose the right answer for each question, while the minimum possible score was zero. The mean (standard deviation, S.D.) and median of total score for each surveyed caregivers before/after seminar was calculated. As the vaccination knowledge variables and the total score of the vaccination knowledge were non-parametric distributions, we used the Wilcoxon signed ranks test for continuous data, and used the McNemar *χ^2^* test for categorical data to compare the difference of vaccination knowledge before and after the health education seminar.

In order to explore the potential socio-demographic determinants of caregivers’ baseline vaccination knowledge level, we applied the Kruskal-Wallis test and Mann-Whitney test for the univariable analysis. All variables that were significantly associated with the knowledge level (*p* < 0.10) in univariable analysis were included into the multivariable analysis. Single-level logistic regression analysis was adopted to obtain adjusted odds ratio (AOR) with 95% *CI*. The caregivers’ baseline vaccination knowledge scores were dichotomized as “≥7 points or <7 points”. All the analysis applied above were completed with Statistics Package for Social Science (SPSS) software, version 13.0 (SPSS Inc, Chicago, IL, USA).

### 2.6. Ethical Considerations

This study was approved by the Ethical Review Board of Zhejiang Provincial Center for Disease Control and Prevention. All the caregivers who agreed to attend the seminar needed to read and sign an informed consent form before they were involved in this study. Participation was voluntary and all the responses were anonymous.

## 3. Results

### 3.1. Socio-Demographic Characteristics

A total of 378 caregivers agreed to participate in our study and attended the health education seminar on vaccination from June to July 2014. Actually, there were 6238 children registered in the 13 vaccination clinics in the same period in Yiwu and the participation rate was only 6.1%. The majority of caregivers were mothers (87.0%) and 58.2% of surveyed caregivers were 20–30 years of age. The education level of 60.1% of surveyed caregivers was senior school; 73.3% of surveyed caregivers were migrant; 50.8% of surveyed caregivers had two preschool children; 45.8% of the surveyed caregivers lived in rural place; 57.1% of surveyed caregivers had no jobs; and 69.6% of the surveyed family earned more than 5000 RMB per month (Table 1).

**Table 1:** Socio-demographic characteristics of the surveyed caregivers (N = 378).

Variables	Level	Frequency	
		No.	%
Age			
	<20	47	12.4
	20–30	220	58.2
	>30	111	29.4
Gender			
	Female	329	87.0
	Male	49	13.0
Education level			
	≤ Junior school	57	15.1
	Senior school	227	60.1
	College	94	24.9
Immigration status			
	Migrant	277	73.3
	Resident	101	26.7
No. of preschool children			
	1	130	34.4
	2	192	50.8
	≥ 3	56	14.8
Living place			
	Rural	173	45.8
	Urban	205	54.2
Employment status			
	Employed	162	42.9
	Unemployed	216	57.1
Household income per month			
	< 5000 RMB	115	30.4
	≥ 5000 RMB	263	69.6

### 3.2. Scores of Vaccination Knowledge

The knowledge assessment results before and after the health education seminar are presented in Table 2. The correct response rates had increased for all ten questions after the health education seminar. Of the ten questions, the pre- and post-seminar correct response rates for nine questions were significantly different. The surveyed caregiver’s total score of vaccination knowledge before and after seminar was compared based on the number of questions answered correctly. The mean total score for the assessment before the seminar was 5.2 ± 1.2 while that was 8.4 ± 0.9 for the assessment after the seminar, with a significant increase of 3.18 points (Table 3).

In univariate analysis for exploring the determinants of caregivers’ baseline vaccination knowledge level, we found that the caregivers’ knowledge level was significantly associated with their education level, immigration status, employment status and household income per month (Table 4). In the single-level logistic regression analysis, we found that caregivers’ education level, immigration status, and household income per month still remained in the final model (Table 5).

**Table 2:** Comparison of caregivers’ knowledge level on vaccination before and after health education seminar (N = 378).

Questions	Correct Response [n (%)]		*χ^2^*	*p*
	Before Seminar	After Seminar		
The immunization program of China include 9 vaccines and 22 doses	57 (15.1)	305 (80.7)	109.3	<0.001
Vaccination of EPI is free for all children	126 (33.3)	328 (86.8)	40.2	<0.001
Active immunization is a killed or weakened form a specific pathogen	17 (4.5)	262 (69.3)	82.2	<0.001
The vaccination of children started at birth	253 (66.9)	347 (91.8)	9.2	0.022
The immunity could be achieved without vaccination	229 (60.6)	332 (87.8)	13.8	0.010
Different vaccines can be administered simultaneously	73 (19.3)	275 (72.8)	43.8	<0.001
Vaccine should not be administered in some unhealthy situations	293 (77.5)	329 (87.0)	3.4	0.102
Some vaccines need more than one dose to get the complete protection	164(43.4)	310(82.0)	15.4	0.008
Side effects of vaccination always are serious and life-threatening	95(25.1)	273(72.2)	32.8	<0.001
The interval period of two vaccination doses is at least four weeks	42(11.1)	296(78.3)	143.4	<0.001

**Table 3:** Comparison of the total score of vaccination knowledge of surveyed caregivers before and after the health education seminar.

Variables	Mean	S.D.	Median	Minimum	Maximum	*Z*	*p*
Knowledge score						−3.869	<0.001
Before seminar	5.2	1.2	6	2	9		
After seminar	8.4	0.9	8	7	10		

**Table 4:** Caregivers’ socio-demographic characteristics and their baseline total score of vaccination knowledge.

Variables	Level	Total Score of Vaccination		*Z*	*p*
		Mean	Median		
Age				−0.436	0.327
	<20	6.2	5.5		
	20–30	6.1	5.5		
	>30	5.8	5		
Gender				−0.068	0.945
	Male	5.6	5		
	Female	7.2	6.5		
Education level				−4.147	<0.001
	≤ Junior school	4.5	4		
	Senior school	6.7	5.5		
	College	7.9	7		
Immigration status				−3.577	<0.001
	Migrant	5.3	5		
	Resident	7.5	6.5		
No. of preschool children				−0.588	0.556
	1	5.6	5		
	2	6.3	5.5		
	≥3	6.7	6		
Living place				−0.816	0.375
	Rural	6.2	5		
	Urban	7.1	6		
Employment status				−2.006	0.045
	Employed	5.8	5		
	Unemployed	7.3	6		
Household income per month				-	
	<5000 RMB	5.1	4.5	−3.962	<0.001
	≥5000 RMB	7.7	6		

**Table 5:** Multivariate analysis for caregivers’ baseline vaccination knowledge scores.

Variables	Level	*p*	AOR(95%*CI*)
Education level			
	≤ Junior school ^*^	-	-
	Senior school	<0.001	1.64(1.12–2.33)
	College	<0.001	2.35(1.38–4.17)
Immigration status			
	Migrant ^*^	-	-
	Resident	<0.001	4.65(2.91–7.60)
Household income per month			
	<5000 RMB ^*^	-	-
	≥5000 RMB	<0.001	3.68(1.98–6.03)

Note: *: reference.

## 4. Discussion

According to the socio-demographic characteristics of the surveyed caregivers, mothers constituted the majority of the participants. Our finding implicated that childhood vaccination was the responsibility of the mothers under most situations, rather than fathers. It was well reported that caregivers’ knowledge level had a significant influence on children’s vaccination coverage rate and timeliness of vaccination worldwide [8,11,23]. In this one group pre and post-assessment design study, a significant increase in caregivers’ knowledge on vaccination was found compared with the baseline level, which demonstrated that the one-hour education seminar was an effective way to improve the vaccination knowledge level of caregivers. As vaccination providers are sometimes the main source of information on immunization for caregivers, it is important that they understand caregivers’ knowledge on vaccination and familiarize themselves with different socio-demographic background of caregivers to remain update to date about the issues of vaccination hesitancy [24].

Our study found some socio-demographic characteristics of the caregivers that were associated with their baseline vaccination knowledge. Consistent with previous reports [25], migrant caregivers had lower overall knowledge on vaccination in this study. We assumed that migrant people had a poor awareness of health and may have already enjoyed some social support, would avail themselves of vaccination services better. Our study revealed that caregivers with lower education level or household income per month had poorer knowledge on vaccination than those with higher education level or monthly household income. These findings were consistent with previous reports [18,26,27]. We assumed that caregivers with higher education level may have a better understanding of knowledge on vaccination and households with a better monthly income may free the caregivers from the struggle of doing more work to survive.

Our study indicated that health education intervention designed for caregivers, such as seminars, could have important implication for improving the awareness and knowledge of vaccination. These finding are consistent with similar previous reports. A German study indicated that using health information leaflets increased girls’ and their parents’ knowledge of the human papillomavirus vaccine and the coverage rate [28]. Caregivers of Guatemala revealed that workshops at the community level were one of the best ways to improve their awareness and knowledge level on immunization [29].

As far as we know, our study was the first to evaluate the effectiveness of a short education seminar on vaccination and caregivers’ concerns about vaccination in Yiwu and Zhejiang province. This study indicated that one-hour education seminars given to caregivers in vaccination clinics were an effective and practical strategy to improve the knowledge level on vaccination. Furthermore, this study provided data on caregivers’ knowledge of vaccination, which generated the baseline level for improving the current immunization coverage rate. Our findings can enable policy makers to develop a short, community-based, health education program at vaccination clinics, especially for migrant caregivers, caregivers with lower education level and lower household income.

This study was subjected to several limitations. First, a pre- and post-assessment without follow up could not evaluate the long-term effectiveness of this intervention on immunization coverage rate. Second, the study was implemented only with caregivers from Yiwu, and the findings should not be extrapolated to caregivers from other areas. Third, there would be selection bias due to the study setting only including vaccination clinics and only 6% of the caregivers participated in the seminars. These limitations should be addressed in future researches.

## 5. Conclusions

The health education intervention adopted in this study focused on improving the vaccination knowledge level of caregivers in Yiwu and made a remarkable increase in their vaccination knowledge compared with the baseline level. Further studies using a large, random sample from other areas and a long-term follow up are needed to evaluate the actual effectiveness of this intervention in improving caregivers’ vaccination knowledge, and also to explore the cost-effectiveness of such an intervention.
